# The Role of Calcitonin Gene‐Related Peptide in High‐Altitude Headache: A Prospective Field Study

**DOI:** 10.1002/acn3.70374

**Published:** 2026-03-30

**Authors:** Roman Schniepp, Ludwig Karrasch, Helena Breitenstein, Andreas Straube, Katharina Kamm

**Affiliations:** ^1^ Department of Neurology University Hospital, LMU Munich Munich Germany; ^2^ Institut für Notfallmedizin und Medizinmanagement (INM) University Hospital, LMU Munich Munich Germany; ^3^ Department of Anaesthesiology and Intensive Care Klinikum Garmisch‐Partenkirchen Garmisch‐Partenkirchen Germany

**Keywords:** acute mountain sickness, calcitonin gene‐related peptide, high‐altitude headache, hypoxia, migraine, tear fluid

## Abstract

**Objective:**

High‐altitude headache (HAH) is a common neurological condition associated with rapid ascent to high altitude. The pathophysiological mechanisms underlying HAH remain incompletely understood. Calcitonin gene‐related peptide (CGRP), a neuropeptide implicated in migraine pathophysiology, may play a key role in the pathophysiology of the disorder due to the shared clinical presentation of migraine and HAH. This study investigated tear fluid CGRP concentrations in response to high‐altitude exposure and headache in healthy volunteers.

**Methods:**

A prospective field study was conducted during a high‐altitude trekking expedition in the Alps. Headache and accompanying symptoms were raised using a structured questionnaire, and tear fluid samples were collected at study Day 1 (baseline, 450 m), at study Day 3 (3647 m), and at study Day 4 (4554 m). Tear fluid CGRP concentrations were quantified using a commercial ELISA kit.

**Results:**

Thirteen participants were included in the analysis. At 4554 m, 61.5% of participants reported a HAH. In participants with HAH, increase of tear fluid CGRP was significantly higher compared to participants without HAH (HAH: +1.49 ± 1.74 ng/mL, no HAH: −0.42 ± 0.64 ng/mL; *p* = 0.011). Increase of tear fluid CGRP levels was significantly higher in participants with a moderate‐to‐severe headache (+1.86 ± 1.86 ng/mL) compared to participants with mild headache (−0.19 ± 0.75 ng/mL; *p* = 0.008).

**Interpretation:**

The results support a potential role for CGRP in the pathophysiology of HAH. Further studies with a larger number of participants are needed to determine the mechanisms of the disorder.

## 1 Introduction

Headache is a common symptom at high altitude that can occur as high‐altitude headache (HAH) or as a symptom of acute mountain sickness (AMS) [1, 2]. HAH is a prevalent neurological condition arising when individuals ascend rapidly above 2500 m without sufficient acclimatization [3, 4]. According to the International Classification of Headache Disorders, 3rd edition (ICHD‐3), HAH is characterized by a bilateral, mild‐to‐moderate headache aggravated by physical exertion and typically occurring within 24 h following ascent above 2500 m (see Table 1A for ICHD‐3 criteria of HAH) [5]. Hypobaric hypoxia and associated changes in cerebral blood flow, intracranial pressure, and mechanisms of cerebral autoregulation are hypothesized to contribute to the pathophysiology of HAH. However, the underlying pathophysiological mechanisms remain to be understood [2, 6, 7].

**TABLE 1:** International Classification of Headache Disorders, 3rd edition [5].

**A. High‐altitude headache**			
A.	Headache fulfilling criterion C		
B.	Ascent to altitude above 2500 m has occurred		
C.	Evidence of causation demonstrated by at least two of the following:		
	1.	Headache has developed in temporal relation to the ascent	
	2.	Either or both of the following:	
		(a)	Headache has significantly worsened in parallel with continuing ascent
		(b)	Headache has resolved within 24 h after descent to below 2500 m
	3.	Headache has at least two of the following three characteristics:	
		(a)	Bilateral location
		(b)	Mild or moderate intensity
		(c)	Aggravated by exertion, movement, straining, coughing and/or bending
D.	Not better accounted for by another ICHD‐3 diagnosis.		

**B. Migraine without aura**		
A.	At least five attacks fulfilling criteria B–D	
B.	Headache attacks lasting 4–72 h (when untreated or unsuccessfully treated)	
C.	Headache has at least two of the following four characteristics:	
	1.	Unilateral location
	2.	Pulsating quality
	3.	Moderate or severe pain intensity
	4.	Aggravation by or causing avoidance of routine physical activity (e.g. walking or climbing stairs)
D.	During headache at least one of the following:	
	1.	Nausea and/or vomiting
	2.	Photophobia and phonophobia
E.	Not better accounted for by another ICHD‐3 diagnosis	

Migraine and HAH share clinical features including exacerbation of symptoms during physical activity [5]. Migraine is a primary headache disorder characterized by recurring moderate‐to‐severe headache attacks, often unilaterally located and of pulsating pain quality. Accompanying symptoms are photo‐ or phonophobia, nausea or vomiting. Many patients report an aggravation by routine physical activity (see Table 1B for ICHD‐3 criteria of migraine) [5]. A history of migraine has been identified as a risk factor for HAH and the prevalence of migraine appears to be greater in individuals residing at elevated altitudes [8, 9, 10]. Further, the occurrence of headache and a migraine‐like headache triggered by hypoxia was shown during the stay at an altitude chamber. Normobaric hypoxia (FiO_2_ = 12.6%) triggered HAH in 81% of healthy volunteers. A migraine‐like headache fulfilling ICHD‐3 criteria occurred in 8% and 15% of participants after 6 and 12 h exposure, respectively [11]. The same study group showed that normobaric hypoxia induced headache in 80% and migraine in 63% of migraine patients after 6 h exposure. Elevated plasma calcitonin gene‐related peptide (CGRP) levels were found during normobaric hypoxia and were significantly higher in patients with migraine without aura [12, 13].

CGRP is a potent vasodilatory neuropeptide released from trigeminovascular endings and plays a central role in migraine pathogenesis [14, 15]. Elevated CGRP levels are observed during migraine attacks and administration of CGRP can provoke a migraine attack in migraine patients [16, 17, 18].

Based on the shared clinical picture of HAH and migraine as well as hypoxia as a trigger for both headache disorders, we hypothesized that the activation of the trigeminovascular system and the subsequent release of the neuropeptide CGRP may also underlie HAH.

The objective of this study was to examine tear fluid CGRP levels in relation to high altitude and the occurrence of headache.

## 2 Materials and Methods

### 2.1 Participants

Participants were recruited via public advertisements (www.ludwig‐karrasch.de). The study was conducted in accordance with the Declaration of Helsinki and approved by the ethics committee of Ludwig‐Maximilians‐University Munich (reference number: 20‐1136). All participants provided written informed consent.

Inclusion criteria comprised healthy adults aged 18–65 years. Exclusion criteria included a history of primary or secondary headache disorders, use of contact lenses on study days, and any pre‐existing neurological, ophthalmological, cardiovascular, or severe psychiatric or systemic medical condition. Study participants were excluded if they stayed overnight at an altitude above 2500 m 4 weeks prior to study inclusion. Pregnant or breastfeeding women were excluded, as were participants with arterial hypertension (≥ 140/90 mmHg), presence of headache or use of abortive headache medication within 48 h prior to baseline assessment.

### 2.2 Study Procedure

**FIGURE 1:**
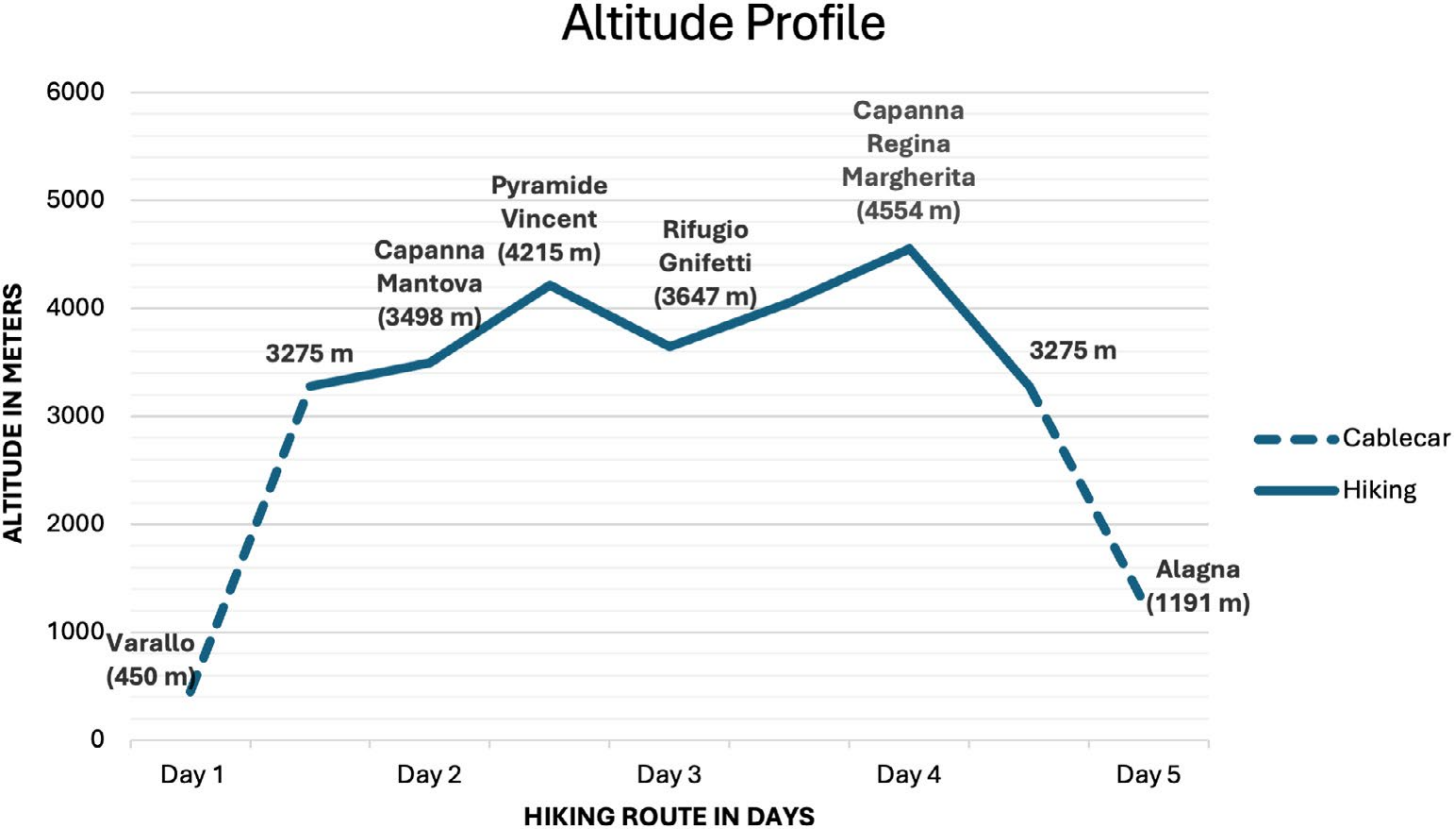
High‐altitude exposure. At study Day 1, participants met at Varallo (450 m) and stayed there overnight. At the morning of study Day 2, the participants and mountain guides went up via cable car and hiking to the Mantova hut (3498 m). The next day, the groups ascended to the Pyramide Vincent (4215 m) and descended to the Gnifetti hut (3647 m, study Day 3). At study Day 4, participants hiked to the Capanna Margherita (4554 m). At study Day 5, participants hiked back to the cable car station at 3275 m and went back to Alagna (1191 m) via cable car.

The study was a prospective, nonrandomized, open‐label field study conducted over 5 consecutive days during a high‐altitude expedition in the Alps in August 2022 (see Figure 1 and Table 2). Participants ascended in groups, accompanied by certified mountain guides (International Federation of Mountain Guides Association, IFMGA). At study Day 1 (baseline), participants met at Varallo (450 m) and after a thorough interview concerning pre‐existing medical conditions and a medical examination, tear fluid was collected from both eyes as described below. At study Day 2, participants and mountain guides ascended to 3275 m via cable car, followed by a 90‐min hike to Capanna Mantova at 3498 m. After a rest period, participants completed a structured headache questionnaire with rating of headache intensity using the numerical rating scale (NRS: 0–10), indication of accompanying symptoms and the 2018 Lake Louise Acute Mountain Sickness Score was raised [19]. At study Day 3, the groups ascended to the Pyramide Vincent (4215 m) and descended to Capanna Giovanni Gnifetti at 3611 m. At study Day 4, the final ascent was performed to Capanna Regina Margherita (4554 m), where participants stayed overnight. At study Days 3 and 4, participants filled in the headache questionnaire and tear fluid was collected from both eyes after a rest period. At Day 5, participants descended by foot and cable car to Alagna (1191 m). The use of personal acute medication was permitted at any time, if needed, and documented.

**TABLE 2:** High‐altitude exposure.

Day	Location	Altitude	Headache questionnaire	LLS‐AMS‐Score	Tear fluid sampling	Medical examination	TCCD sonography
1	Varallo	450 m	x	x	x	x	x
2	Capanna Mantova	3498 m	x	x	—	—	—
3	Rifugio Gnifetti	3647 m	x	x	x	(x)	—
4	Capanna Regina Margherita	4554 m	x	x	x	(x)	x
5	Alagna	1191 m	x	x	—	—	—

*Note:* The table shows the examinations at the respective study days. At study Days 3 and 4, a medical examination was conducted if participants reported symptoms.

Abbreviations: LLS‐AMS‐Score, 2018 Lake Louise Acute Mountain Sickness Score; TCCD, transcranial color‐coded duplex.

### 2.3 Tear Fluid Collection and CGRP Measurement

Tear fluid was collected from both eyes separately after participants rested supine for 5 min as described before [20, 21]. Tear fluid was aspirated at the lateral canthus of both eyes using plastic capillaries (ref. no. 100012, Sanguis, Nümbrecht, Germany) for a maximum of 1 min. Care was taken to avoid ocular irritation and reflex tearing, which may dilute the sample. After tear fluid collection, samples were immediately immersed in chilled 1.5 mL tubes containing 500 μL tissue protein extraction reagent (TPER; Pierce, Rockford, IL), stored on dry ice for up to 3 days, and subsequently frozen at −80°C. Before analysis, samples were centrifuged (4000 g, 5 min). CGRP levels were determined using a commercial ELISA kit (Abbexa [Cambridge, UK]: detection range 3.13–200 pg/mL; sensitivity 1.88 pg/mL). Each sample was measured in duplicate according to manufacturer instructions. Absorbance was read using a spectrometer (PerkinElmer 2030, USA) and concentrations were calculated using a four‐parameter logistic (4PL) curve. Final CGRP values were expressed as the average of the two measurements per sample. No significant differences in tear fluid CGRP concentrations were observed between the right and left eye at baseline (right eye: 1.79 ± 1.69 ng/mL; left eye: 2.09 ± 1.78 ng/mL; Wilcoxon test: *z* = 1.363, *p* = 0.173, *n* = 13), for analysis tear fluid CGRP levels from the right and left eyes were pooled.

### 2.4 Transcranial Color‐Coded Duplex Sonography

Transcranial color‐coded duplex (TCCD) sonography was performed at study Day 1 (baseline) and study Day 4 (4554 m) in a quiet room by the same examiner (L.K.) to avoid inter‐rater variability. Participants were positioned supine. The internal carotid artery and middle cerebral artery were examined bilaterally using a 12 L‐RS Wideband Linear Array and 3Sc‐RS Phased Array probe. The middle cerebral artery was examined at a depth of 40–60 mm over the temporal region. Mean flow velocity (MFV = (PSV + 2×EDV)/3) and Lindegaard Index (LI = MFV_MCA_/MFV_ICA_) were determined [22].

### 2.5 Oxygen Saturation Measurement

Participants wore an in‐ear MDR‐approved pulsoxymetry device (Cosinuss cmed Alpha, Cosinuss GmbH, Munich, Germany) during the tour that delivered photoplethysmographic oxygen saturation (SpO_2_) and heart rate frequency.

### 2.6 Statistics

Data is presented as mean ± standard deviation unless otherwise specified. Nonparametric tests were used since some data was not normally distributed. Headache frequency and AMS were compared over different time points using the Cochran's *Q* test. For the comparison of headache intensity and tear fluid CGRP levels over different points in time, the Friedman test followed by post hoc Dunn‐Bonferroni tests, if appropriate, or the Wilcoxon test was used.

Headache intensity, tear fluid CGRP levels, oxygen saturation or blood flow velocity between different groups (HAH/no HAH, migraine‐like headache/no migraine‐like headache) was analyzed by Mann–Whitney *U* test. Statistical analysis was performed with SPSS 29 (IBM, Corp., Armonk, NY, USA). Significance was accepted at *p* < 0.05 (two‐tailed).

## 3 Results

Of the 24, 11 participants were excluded due to early termination (*n* = 2) or insufficient tear fluid sampling (*n* = 9), resulting in a final sample of 13 participants (male = 8, mean age: 35.1 ± 7.0 years). All participants completed the high‐altitude exposure protocol and provided sufficient tear fluid samples at all required time points.

### 3.1 Headache Incidence and Severity at High Altitude

Participants reported a significant increase in the frequency and severity of headache with increasing altitude exposure. Headache frequency and intensity was highest at study Day 4, reaching 3.2 ± 2.2 at the NRS (Friedman‐test: *χ* ^2^[3] = 26.944, *p* < 0.001, *n* = 13; post hoc Dunn‐Bonferroni test: Day 1 vs. Day 2: *z* = −0.808, *p* = 0.664; Day 1 vs. Day 3: *z* = −1.000, *p* = 0.290; Day 1 vs. Day 4: *z* = −2.346, *p* < 0.001; Day 2 vs. Day 3: *z* = −0.192, *p* = 1.000; Day 2 vs. Day 4: *z* = −1.538, *p* = 0.014; Day 3 vs. Day 4: *z* = −1.346, *p* = 0.047, see Table 3).

**TABLE 3:** Clinical characteristics over the course of the high‐altitude exposure.

	Day 1 (450 m)	Day 2 (3497 m)	Day 3 (3647 m)	Day 4 (4554 m)	
Headache (%)	0 (0%)	6 (46.2%)	8 (61.5%)	11 (84.6%)	*p* < 0.001^b^
NRS (0–10)	0 ± 0	0.7 ± 1.0	1.0 ± 1.0	3.2 ± 2.2	*p* < 0.001^c^
Oxygen saturation, %^a^	—	—	78 ± 7	78 ± 7	*p* = 0.646^d^
Heart rate (bpm)*	—	—	99 ± 26	106 ± 26	*p* = 0.093^d^
High‐altitude headache, *n* (%)	0 (0%)	0 (0%)	2 (15.4%)	8 (61.5%)	*p* < 0.001^b^
Migraine, *n* (%)	0 (0%)	0 (0%)	0 (0%)	6 (46.2%)	*p* < 0.001^b^
Intake of acute medication (%)	0 (0%)	0 (0%)	0 (0%)	1 (7.7%)	—
Acute Mountain Sickness, *n* (%)	0 (0%)	2 (15.4%)	4 (30.8%)	11 (84.6%)	*p* < 0.001^b^
Mild, *n* (%)	—	2 (100%)	3 (75.0%)	8 (72.7%)	
Moderate, *n* (%)	—	—	1 (25.0%)	3 (27.3%)	
Severe, *n* (%)	—	—	—	—	
Lake Louise Score (0–12)	0.8 ± 0.8	1.5 ± 1.2	2.2 ± 1.7	4.1 ± 2.2	*p* < 0.001^c^

*Note:* At each study day, a headache questionnaire with headache symptoms and accompanying symptoms was obtained. Further, the Lake Louise Acute Mountain Sickness Score and the ICHD‐3 criteria for high‐altitude headache and migraine were raised.

^a^ Heart rate and oxygen saturation was measured in 11 participants. bpm beats per minute.

^b^ Cochran's *Q* test.

^c^ Friedman test.

^d^ Wilcoxon test.

The diagnostic criteria for HAH according to the ICHD‐3 criteria were fulfilled in two participants (15.4%) at study Day 3, rising to eight participants (61.5%) at study Day 4 (Cochran's *Q* test: *χ* ^2^ [3, *n* = 13] = 19.846, *p* < 0.001, see Table 4).

**TABLE 4:** High‐altitude headache at 4554 m (study Day 4).

Participant	Criterion B	Criterion C					
		1	2		3		
	Ascent > 2500 m	Headache development in temporal relation to ascent	(a) Worsening of headache with continuing ascent	(b) Resolution of headache within 24 h after descent < 2500 m	(a) Bilateral location	(b) Mild/moderate intensity	(c) Aggravation
1	X	X	X	X	X	X	
3	X	X		X		X	X
4	X	X		X		X	
18	X	X	X	X	X	X	
19	X	X		X	X	X	X
20	X	X	X	X		X	X
21	X	X	X	X			X
23	X	X	X	X	X		X

*Note:* Eight participants fulfilled ICHD‐3 criteria of high‐altitude headache at 4554 m. Here, the characteristics of the headache according to the ICHD‐3 criteria are shown.

AMS based on the 2018 Lake Louise Acute Mountain Sickness Score was reported by two participants (15.4%) at study Day 2, four participants (30.8%) at study Day 3, and 11 participants (84.6%) at study Day 4 (Cochran's *Q* test: *χ* ^2^ [3, *n* = 12] = 19.765, *p* < 0.001). At study Day 4, headache and fatigue were the most often reported symptoms by 90.9% of participants, respectively. 45.5% of participants reported a moderate or intense headache. Gastrointestinal symptoms occurred in 36.4% of participants, and dizziness was reported by 27.3% of participants. Due to the high number of participants with AMS, further group comparisons were not possible.

### 3.2 Tear Fluid CGRP at High Altitude

**FIGURE 2:**
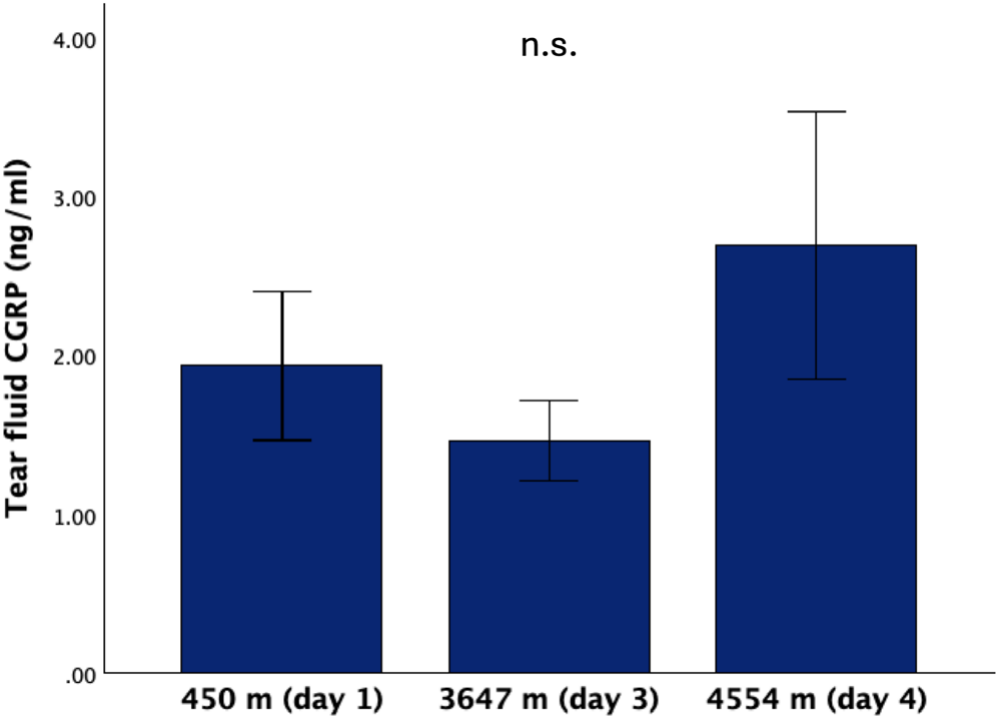
Tear fluid CGRP over the course of the high‐altitude exposure. At study Day 1, 3, and 4 tear fluid samples were obtained. Over all participants, tear fluid CGRP levels were not significantly different over the course of the high‐altitude exposure (*p* = 0.146). n.s. not significant.

Over the course of the high‐altitude exposure, tear fluid CGRP levels at study Day 4 were not significantly different from baseline in the entire study population (Day 1: 1.94 ± 1.69 ng/mL, Day 3: 1.50 ± 0.95 ng/mL, Day 4: 2.69 ± 3.04 ng/mL, Friedman test: *χ* ^2^ (2) = 3.846; *p* = 0.146, *n* = 13; see Figure 2).

### 3.3 Elevated Tear Fluid CGRP Levels Depend on Higher Headache Intensity

**FIGURE 3:**
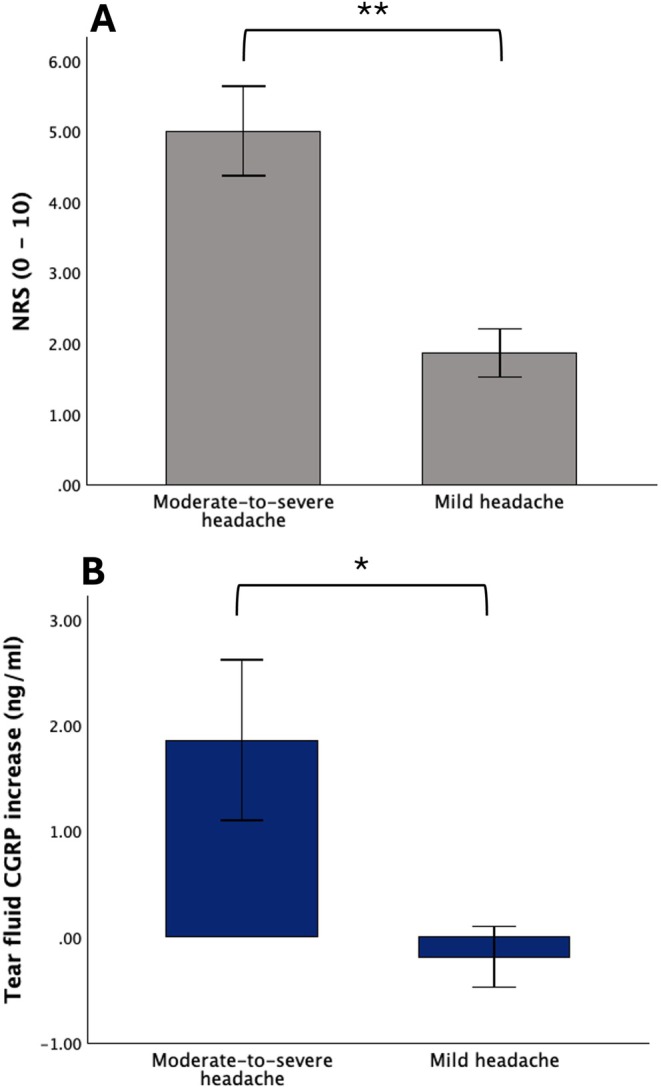
Headache and tear fluid CGRP increase according to headache intensity. (A) Participants were divided due to headache intensity in a group with moderate‐to‐severe headache (mean NRS: 5.0 ± 1.5, *n* = 6) compared to a group with mild headache (mean NRS: 1.9 ± 0.9, *p* < 0.001). (B) Participants with a moderate‐to‐severe headache showed a significantly higher increase of tear fluid CGRP levels at study Day 4 (*p* = 0.008). Bars represent standard error. NRS numerical rating scale, TF tear fluid, **p < 0.001, *p < 0.05.

Six participants reported a moderate‐to‐severe headache with a mean NRS of 5.0 ± 1.5 compared to seven participants with a mild headache at study Day 4 (NRS: 1.9 ± 0.9; Mann–Whitney *U* test: *U* = 42.000, *p* < 0.001; Figure 3A).

Participants with a moderate‐to‐severe headache intensity showed a significant higher rise in tear fluid CGRP levels compared to participants with a mild headache (moderate‐to‐severe headache: +1.86 ± 1.86 ng/mL, mild headache: −0.19 ± 0.75 ng/mL; Mann–Whitney *U* test: *U* = 39.000, *p* = 0.008; Figure 3B). Baseline CGRP levels did not differ significantly between these two groups (moderate‐to‐severe headache: 2.20 ± 2.52 ng/mL, mild headache: 1.71 ± 0.51 ng/mL; Mann–Whitney *U* test: *U* = 14.000, *p* = 0.366).

### 3.4 Increase in Tear Fluid CGRP Is Elevated in High‐Altitude Headache

**FIGURE 4:**
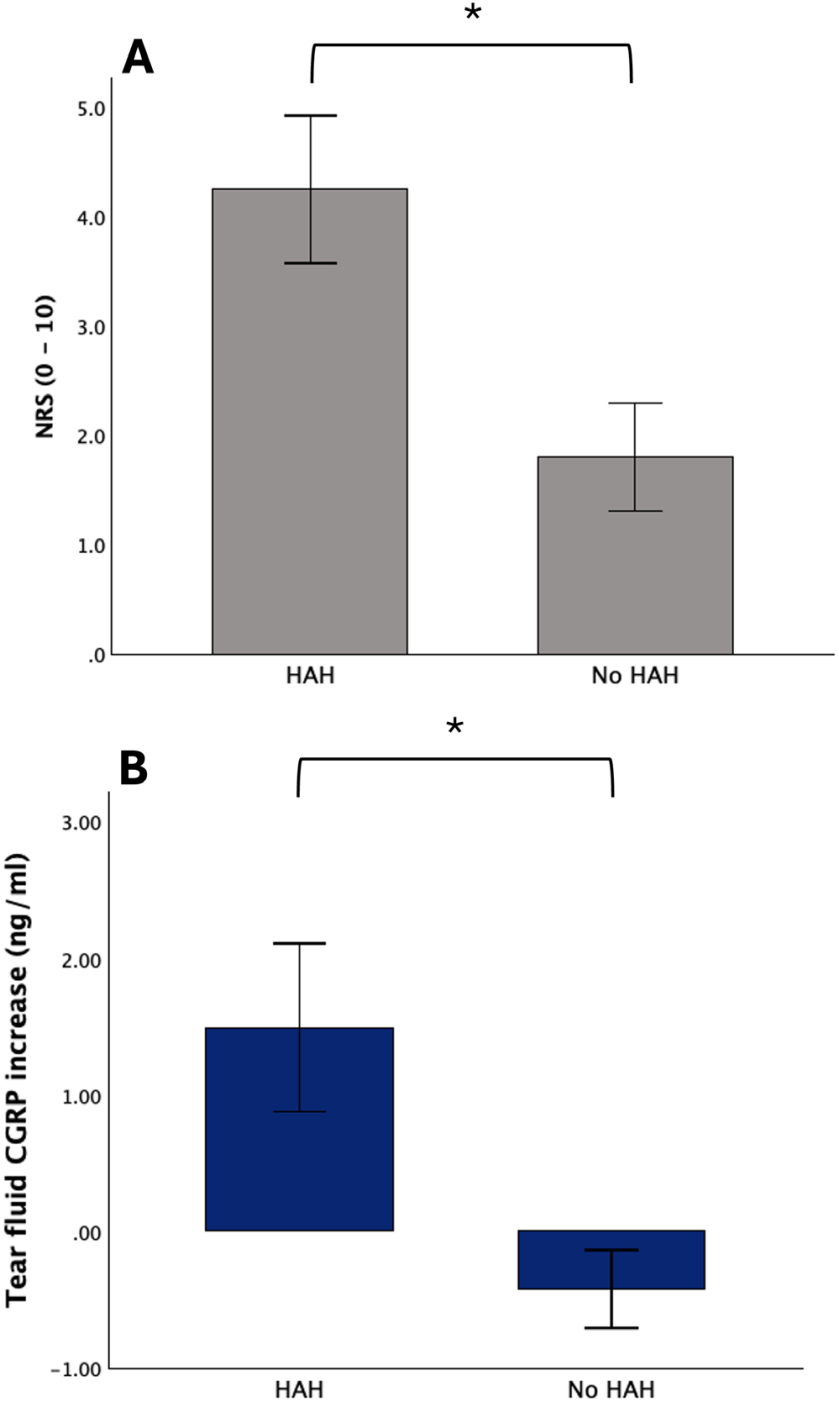
High‐altitude headache at 4554 m. (A) Eight participants reported high‐altitude headache according to the ICHD‐3 criteria at study Day 4. Headache intensity was more intense in the HAH group (*p* = 0.019). (B) The increase of tear fluid CGRP levels was significantly higher in the HAH group compared to participants without HAH at study Day 4 (*p* = 0.011). Bars represent standard error. NRS numerical rating scale, TF tear fluid, HAH high‐altitude headache, * *p* < 0.05.

Eight participants (61.5%) fulfilled ICHD‐3 criteria for HAH at study Day 4. Participants suffering from HAH showed significantly higher headache intensities (mean NRS: HAH: 4.3 ± 1.9, no HAH: 1.8 ± 1.1; Mann–Whitney *U* test: *U* = 36.000, *p* = 0.019, see Table 4 and Figure 4A) and a higher increase in tear fluid CGRP levels (HAH: +1.49 ± 1.74 ng/mL, no HAH: −0.42 ± 0.64 ng/mL; Mann–Whitney *U* test: *U* = 37.000, *p* = 0.011, Figure 4B). There was no significant difference in tear fluid CGRP levels at baseline between the two groups (HAH: 1.99 ± 2.18 ng/mL, no HAH: 1.86 ± 0.43 ng/mL; Mann–Whitney *U* test: *U* = 10.000, *p* = 0.171). Oxygen saturation was not different between these groups at study Day 4 (HAH: 78% ± 6%; no HAH: 79% ± 9%; Mann–Whitney *U* test: *U* = 11.000, *p* = 0.537).

### 3.5 Increase in Tear Fluid CGRP Is Elevated in Migraine‐Like Headache

**FIGURE 5:**
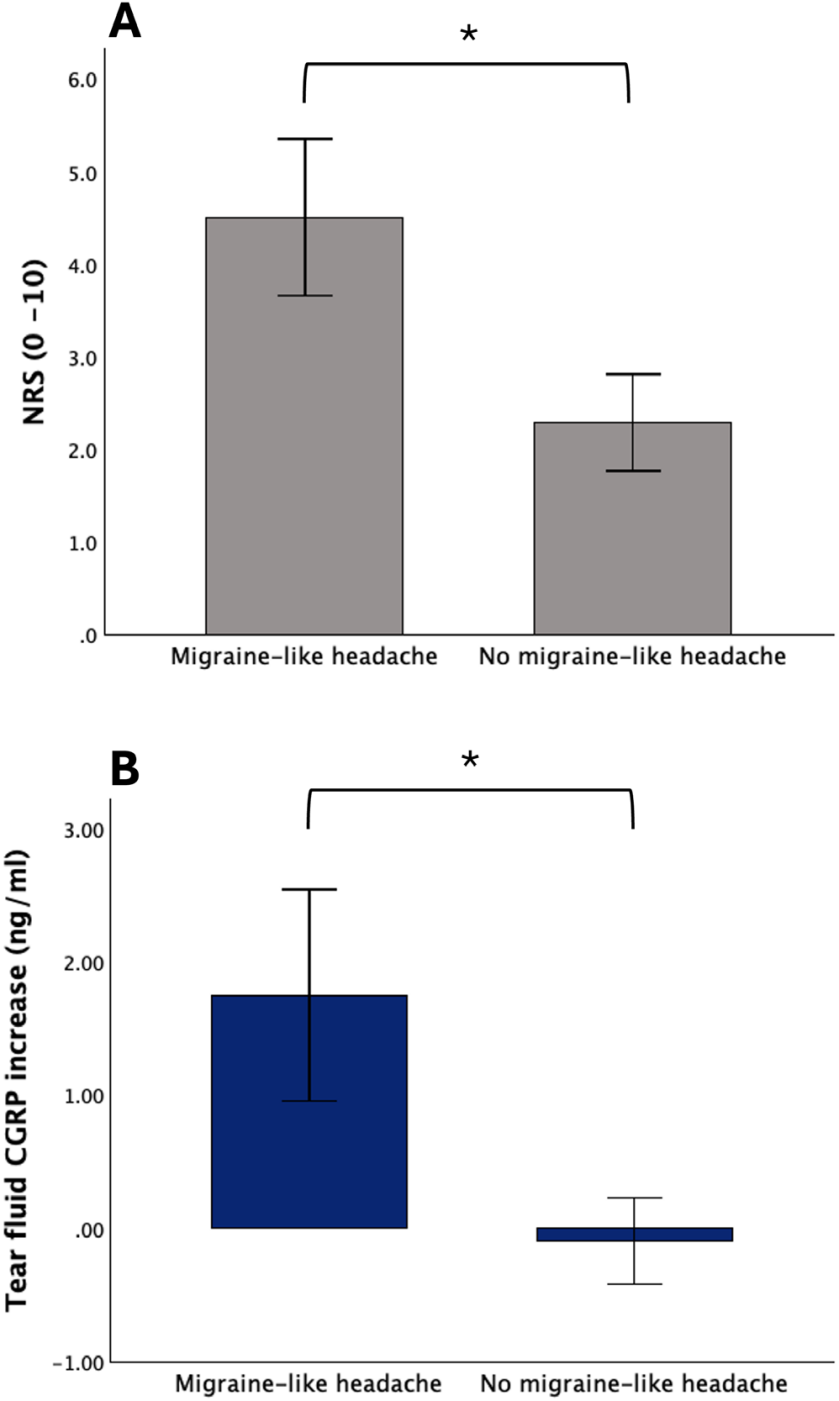
Migraine‐like headache at 4554 m. (A) Six participants reported a migraine‐like headache with a higher headache intensity (*p* = 0.004). (B) The increase of tear fluid CGRP levels was significantly higher in participants with a migraine‐like headache at study Day 4 (*p* = 0.035). Bars represent standard error. * *p* < 0.05, **p < 0.001.

At study Day 4, six participants (46.2%) reported a migraine‐like headache. 83.3% of these participants reported a moderate‐to‐intense headache with aggravation by physical exertion (66.7%). Accompanying symptoms were nausea/vomiting (50.0%) and photophobia (16.7%). These individuals reported a higher headache intensity (mean NRS: migraine‐like headache: 4.8 ± 1.9) than those without migraine features (mean NRS: 1.9 ± 1.2; Mann–Whitney *U* test: *U* = 30.000, *p* = 0.004, Figure 5A). Consistent with this, tear fluid CGRP increased significantly greater in the migraine‐like headache group (+1.75 ± 1.94 vs. −0.09 ± 0.86 ng/mL; Mann–Whitney *U* test: *U* = 36.000, *p* = 0.035, Figure 5B). There was no significant difference in tear fluid CGRP levels at baseline between the two groups (migraine‐like headache: 2.24 ± 2.50 ng/mL; no migraine‐like headache: 1.68 ± 0.54 ng/mL; Mann–Whitney *U* test: *U* = 14.000, *p* = 0.366). There was no difference in oxygen saturation between the two groups at study Day 4 (migraine‐like headache: 75% ± 7%; no migraine‐like headache: 81% ± 6%; Mann–Whitney *U* test: *U* = 8.000, *p* = 0.247).

### 3.6 Cerebral Blood Flow

MCA velocity or changes from baseline in blood flow was not different between participants with HAH or no HAH (right MCA: HAH: 90 ± 19 cm/s; no HAH: 74 ± 14 cm/s; Mann–Whitney *U* test: *U* = 30.000, *p* = 0.171. Left MCA: HAH: 91 ± 24 cm/s; no HAH: 84 ± 22; Mann–Whitney *U* test: *U* = 20.000, *p* = 1.000, see Table 5) or participants with a migraine‐like headache or no migraine‐like headache at study Day 4 (right MCA: migraine‐like headache: 90 ± 21 cm/s; no migraine‐like headache: 78 ± 15 cm/s; Mann–Whitney *U* test: *U* = 27.000, *p* = 0.445. Left MCA: migraine‐like headache: 89 ± 31 cm/s; no migraine‐like headache: 88 ± 15 cm/s; Mann–Whitney *U* test: *U* = 30.000, *p* = 0.171; see Table 5). There was no evidence for vasospasm in all groups (see Table 5).

**TABLE 5:** Cerebral blood flow of the right‐ and left‐sided MCA according to the occurrence of high‐altitude headache or a migraine‐like headache at study Day 4.

		High‐altitude headache	No high‐altitude headache		Migraine‐like headache	No migraine‐like headache	
RMCA	MFV (cm/s)	90 ± 19	74 ± 14	*P* = 0.171	90 ± 21	78 ± 15	*p* = 0.445
	Lindegaard Index	1.8 ± 0.5	1.5 ± 0.3	*p* = 0.284	1.7 ± 0.5	1.6 ± 0.4	*p* = 0.731
	% Baseline	126.7 ± 33.5	99.4 ± 10.6	*P* = 0.093	124.0 ± 40.4	109.4 ± 17.0	*p* = 1.000
LMCA	MFV (cm/s)	91 ± 24	84 ± 22	*P* = 1.000	89 ± 31	88 ± 15	*p* = 0.836
	Lindegaard Index	1.6 ± 0.6	1.5 ± 0.2	*p* = 0.524	1.6 ± 0.7	1.6 ± 0.2	*p* = 0.101
	% Baseline	122.2 ± 21.5	115.8 ± 27.7	*P* = 1.000	107.8 ± 28.2	130.0 ± 12.0	*p* = 0.101

*Note:* There was no significant difference in MFV or changes in blood flow velocity of both‐sided MCA in participants with or without a high‐altitude headache or with or without a migraine‐like headache. Further, there was no evidence for vasospasms in either of the groups.

Abbreviations: MCA, middle cerebral artery; MFV, mean flow velocity.

## 4 Discussion

This prospective field study sought to elucidate whether tear fluid CGRP concentrations rise with increasing altitude and whether such elevations correlate with HAH. Our results revealed a significant elevation of tear fluid CGRP at very high altitude (4554 m) in participants with moderate‐to‐severe headache.

### 4.1 Symptom Profile and Clinical Parallels With Migraine

Headache is frequently observed during rapid ascent to altitude; however, headache symptomatology differs. Previously, three different types of headaches were proposed: as the main symptom of AMS, HAH due to hypoxia (without any AMS symptoms), and a migraine‐like headache triggered by altitude [4].

In this study sample, 61.5% of participants experienced headache fulfilling the diagnostic criteria for HAH at 4554 m. This is consistent with earlier studies, finding 35.6%–73% of participants who reported HAH at an altitude of 4000–4999 m [10, 23, 24, 25].

Six participants (46.2%) reported symptoms consistent with a migraine‐like headache, including moderate‐to‐severe pain intensity, pulsating quality, nausea, and photophobia. These findings align with prior reports describing considerable symptomatic overlap between migraine and HAH. The occurrence of severe headache was reported by 23%–35% of participants in earlier studies [23, 24]. One study found that 16% of participants reported a pulsating headache quality and 11% of participants reported photo‐ and phonophobia [24]. Another study investigated the occurrence of HAH, AMS, or migraine in 667 mountaineers after the descend from Mount Gray/Torreys (4349 m). Forty‐one percent of all hikers experienced headache; of these, 98% reported HAH, 10% met criteria for migraine, and 26% were diagnosed with AMS [10]. The occurrence of a migraine‐like headache was further investigated and corroborated using a normobaric, hypoxic chamber (FiO_2_ = 12.6%). In this study, a migraine‐like headache was induced in 8% and 15% of healthy participants after 6 and 12 h exposure, respectively [11]. The lower incidence of a migraine‐like headache in the above‐mentioned studies compared to our study might be due to shorter time of exposition. The increasing frequency and intensity of headache with ascending altitude supports the notion that high‐altitude exposure provokes headache that may mimic or even trigger migraine‐like attacks. Taken together our data and data from previous studies, it might be speculated that HAH describes a continuum beginning with a low or moderate headache and continuing to severe headache resembling a migraine‐like headache.

### 4.2 Calcitonin Gene‐Related Peptide and Hypoxia

Hypoxia may act as a trigger for the development of headache. For example, normobaric hypoxia (induced in an altitude chamber, FiO_2_ = 12.6%) can trigger headache and a migraine‐like headache in healthy people without a history of a primary headache disorder [11]. In line with this, hypoxia occurred in the study participants, although no difference was found between the participants in terms of headache.

CGRP is a key effector in the pathophysiology of migraine. Released from trigeminovascular afferents, CGRP acts as a potent vasodilator and proinflammatory mediator and induces headache. Elevated CGRP concentrations have been detected during spontaneous and experimental migraine attacks, and intravenous administration of CGRP can induce migraine‐like symptoms in migraine patients [16, 17, 18, 26]. Further, some evidence suggests that CGRP might also play a role in secondary headache since elevated serum CGRP levels have also been detected in posttraumatic and dialysis headache [27, 28].

In our study, tear fluid CGRP concentrations increased significantly with the occurrence of a moderate‐to‐severe headache during the high‐altitude exposure. This result supports the idea that CGRP release is involved in the pathophysiology of HAH and might be part of the pathophysiological cascade underlying headache in general. In this respect, primary and secondary headaches might share a common pathophysiological mechanism. Notably, baseline CGRP levels did not differ between headache subgroups, suggesting that the observed elevations were triggered by altitude‐related stressors rather than constitutive individual variation.

While the exact mechanism remains uncertain, it is plausible that hypoxia and barometric changes at high altitude activate the trigeminovascular system, thereby increasing CGRP secretion. These neurovascular responses may be further amplified in susceptible individuals, potentially explaining the higher CGRP levels in those with more severe headache symptoms.

Evidence for the activation of the trigeminovascular system by hypoxia stems from a study showing elevated plasma CGRP levels in patients with episodic migraine during a 6‐h hypoxic exposure (FiO_2_ = 12.6%) [13]. Barometric changes, like weather changes, are believed to cause primary headaches. However, studies investigating barometric changes in primary headaches are inconsistent, the relevance of barometric changes in the generation of HAH and headache attributed to airplane travel is well described [29]. Trigeminal activation caused by barometric changes has been shown in an animal study. In this study, rats were placed in a climate chamber before, during and after lowering the barometric pressure about 40 hPa and activity of neurons in the spinal trigeminal nucleus was recorded. During the low atmospheric pressure, especially neurons with afferents from the cornea showed an increased activity. The authors concluded that this activation may contribute to headache generation [30]. The influence of small barometric changes was further investigated in a human study. Craniofacial symptoms were raised in 15 subjects without a known primary headache disorder during the lowering of barometric pressure of 20 or 40 versus 0 hPa. The subjects were investigated in a weather chamber and exposed to lowering and low barometric pressure for 24 min. All participants reported head compression and 20% of participants reported a mild or moderate headache during lower barometric pressure [31]. Taken together, there is evidence, that both—barometric changes and hypoxia—may activate the trigeminovascular system. Due to our study design, it is difficult to discriminate between the barometric or hypoxic influence. However, our data suggest that hypoxia alone does not change CGRP levels since we did not find changes in tear fluid CGRP over the course of the high altitude stay. Further, there were no differences in oxygen saturation at study Day 4 between participants with a HAH or a migraine‐like headache compared to participants without. In future, it would be interesting to investigate the two conditions separately in an altitude chamber to differentiate between the respective influence.

Participants with HAH or with a migraine‐like headache or without showed no differences in cerebral blood flow measured bilaterally in the MCA. Similarly, previous studies did not find differences in blood flow velocity in association with AMS [32, 33, 34]. Further, there was no evidence for vasospasms in these groups underlining the activation of the trigeminovascular system.

To the best of our knowledge, this is the first study showing elevated tear fluid CGRP levels due to high altitude. Two recent studies investigated CGRP in relation to hypoxia showing inconsistent results; both studies found no elevation in serum CGRP levels [35, 36]. CGRP levels didn't differ before and after a 9‐h exposure to normobaric hypoxia (12.9% O_2_) in 10 healthy participants [35]. The other study investigated 12 healthy volunteers before and during a 5‐day stay at 4554 m showing no significant differences in serum CGRP levels [36]. However, these studies investigated CGRP in relation to hypoxia rather than headache, and study data should only be compared cautiously since different processing and analyzing methods have been used [37].

### 4.3 Limitations

This study has several limitations. First, the sample size was small (*n* = 13), and nearly half of the original cohort were excluded due to premature termination or insufficient tear fluid collection. This limits statistical power and generalizability.

Second, while tear fluid offers practical advantages, its comparability with plasma or cerebrospinal fluid concentrations of CGRP remains insufficiently validated. Third, the observational design precludes causal inference; it remains uncertain whether CGRP elevation precedes symptom onset or reflects a downstream effect of headache severity.

Future research should focus on larger, controlled studies incorporating multiple biological media.

## 5 Conclusion

This study indicates that headache during exposure to high altitude is associated with a significant increase in tear fluid CGRP levels, particularly in individuals who develop a moderate‐to‐severe headache or symptoms consistent with migraine. The rise in tear fluid CGRP levels with increasing altitude, peaking at 4554 m, supports the hypothesis that hypobaric hypoxia acts as a physiological trigger for trigeminovascular activation and CGRP release.

The close association between CGRP elevation and symptom intensity highlights its potential role in the pathogenesis of HAH. Furthermore, the presence of migraine‐like features in nearly half of participants suggests that altitude may provoke a phenotype that closely resembles primary headache disorders—possibly via shared pathophysiological mechanisms. Interventional studies exploring CGRP‐targeted therapies for HAH prophylaxis may also be warranted.

Tear fluid analysis emerges as a feasible, noninvasive method for neuropeptide quantification in remote or field‐based studies. With further validation, CGRP could serve not only as a biomarker for headache severity but also as a therapeutic target for prophylactic intervention in individuals ascending to high altitude.

## Author Contributions

R.S. study concept and design, critical revision of the manuscript for important intellectual content, supervision. L.K. study concept and design, acquisition of data, analysis (including statistical analyses), drafting the manuscript. H.B. acquisition of data, drafting the manuscript. A.S. critical revision of the manuscript for important intellectual content, supervision. K.K. study concept and design, acquisition of data, analysis (including statistical analyses) and interpretation, drafting the manuscript.

## Funding

The authors have nothing to report.

## Conflicts of Interest

R.S. has received travel grants and/or honoraria from Rölke Pharma, Novartis and Cosinuss GmbH. L.K. has received a grant from BExMed (German Society for Mountain and Expedition Medicine). H.B. has nothing to declare. A.S. has received travel grants and/or honoraria from Allergan/AbbVie, Hormosan, Lilly, Lundbeck, Novartis, Sanofi and Teva. K.K. has received travel grants and/or honoraria from Lundbeck, TEVA, Novartis and IHS.

## Data Availability

Raw data were generated at LMU. Derived data supporting the findings of this study are available from the corresponding author on request.
